# PrismEXP: gene annotation prediction from stratified gene-gene co-expression matrices

**DOI:** 10.7717/peerj.14927

**Published:** 2023-02-27

**Authors:** Alexander Lachmann, Kaeli A. Rizzo, Alon Bartal, Minji Jeon, Daniel J. B. Clarke, Avi Ma’ayan

**Affiliations:** Pharmacological Sciences, Icahn School of Medicine at Mount Sinai, New York, USA

**Keywords:** Transcriptomics, Gene expression, Gene function predictions, RNA-seq, Unsupervised learning, Druggable genome

## Abstract

**Background:**

Gene-gene co-expression correlations measured by mRNA-sequencing (RNA-seq) can be used to predict gene annotations based on the co-variance structure within these data. In our prior work, we showed that uniformly aligned RNA-seq co-expression data from thousands of diverse studies is highly predictive of both gene annotations and protein-protein interactions. However, the performance of the predictions varies depending on whether the gene annotations and interactions are cell type and tissue specific or agnostic. Tissue and cell type-specific gene-gene co-expression data can be useful for making more accurate predictions because many genes perform their functions in unique ways in different cellular contexts. However, identifying the optimal tissues and cell types to partition the global gene-gene co-expression matrix is challenging.

**Results:**

Here we introduce and validate an approach called PRediction of gene Insights from Stratified Mammalian gene co-EXPression (PrismEXP) for improved gene annotation predictions based on RNA-seq gene-gene co-expression data. Using uniformly aligned data from ARCHS4, we apply PrismEXP to predict a wide variety of gene annotations including pathway membership, Gene Ontology terms, as well as human and mouse phenotypes. Predictions made with PrismEXP outperform predictions made with the global cross-tissue co-expression correlation matrix approach on all tested domains, and training using one annotation domain can be used to predict annotations in other domains.

**Conclusions:**

By demonstrating the utility of PrismEXP predictions in multiple use cases we show how PrismEXP can be used to enhance unsupervised machine learning methods to better understand the roles of understudied genes and proteins. To make PrismEXP accessible, it is provided *via* a user-friendly web interface, a Python package, and an Appyter. AVAILABILITY. The PrismEXP web-based application, with pre-computed PrismEXP predictions, is available from: https://maayanlab.cloud/prismexp; PrismEXP is also available as an Appyter: https://appyters.maayanlab.cloud/PrismEXP/; and as Python package: https://github.com/maayanlab/prismexp.

## Introduction

Gene annotations can be automatically inferred by combining established annotations with patterns of mRNA co-expression based on the concept of guilt-by-association. It is generally accepted that genes and proteins that co-express may be functionally related (Choi et al., 2005; Horan et al., 2008; Ala et al., 2008). Proteins with high mRNA expression correlations, or high mutual information (Lachmann et al., 2016), are often found in the same macromolecular complexes, cell signaling pathways, and metabolic circuits (Aoki, Ogata & Shibata, 2007; Zhang, Guo & Xie, 2020). Since proteins predominantly function through the formation of protein complexes and can be found in multiple different complexes, their activity depends on their expression in different cellular and tissue contexts. Tight control of protein complex stoichiometry is essential for their proper formation (Kirkpatrick et al., 2020). While all cells and tissues perform general housekeeping processes, they also have specialized roles and therefore differ in the composition of their pathways and complexes. Due to cell and tissue specificity, it is important to consider gene annotations in their correct cellular and tissue contexts (Sonawane et al., 2017). Thus, reverse-engineered regulatory mechanisms from tissue-agnostic data lacks the details needed to capture gene-gene and protein-protein interactions accurately. With the availability of large compendiums of uniformly aligned RNA-seq data from resources such as ARCHS4 (Lachmann et al., 2018), Genotype-Tissue Expression (GTEx) (Consortium, 2015), or The Cancer Genome Atlas (TCGA) (Weinstein et al., 2013), which include cell type and tissue-specific gene expression information, it is possible to use context-specific gene expression patterns for improve gene annotation predictions.

Over decades of biochemistry, pharmacology, molecular and cell biology research, many mammalian genes have been thoroughly characterized. However, there are still many genes that remain obscured due to a lack of research interest and as a result of focus research biases. Extensive known gene annotations can be accessed from resources such as the Gene Ontology (Ashburner et al., 2000), the Human Phenotype Ontology (Köhler et al., 2017), UniProt (Consortium, 2019), Harmonizome (Rouillard et al., 2016) and Enrichr (Kuleshov et al., 2016). Such gene annotations can be represented as sets of genes associated with a particular biological term such as a disease, a function, or a pathway. Our assumption is that these gene sets are generally incomplete, reflecting our partial knowledge about the involvement of genes and their protein products in biological, physiological, and pathophysiological processes.

Computational methods that use known gene annotations to impute annotations for understudied genes are a useful way for generating hypotheses that can lead to new discoveries. High-content genome-wide data, such as RNA-seq, includes information about under-studied genes and is not biased towards the well-studied genes. Significant efforts are made to produce data, and develop tools, to shed light on understudied genes, particularly those genes that can serve as drug targets (Park et al., 2013; Nariai, Kolaczyk & Kasif, 2007). One such effort is the NIH Common Fund Program Illuminating the Druggable Genome (IDG) (Oprea, 2019). IDG focuses on understudied proteins from the three families with most known drug targets: protein kinases, G-protein coupled receptors (GPCRs), and ion channels. In previous work, we have developed methods to predict gene annotations for all human genes, including members of the protein kinases, GPCRs, and ion channels families, using gene-gene co-expression data from thousands of RNA-seq samples that are tissue and cell type agnostic (Lachmann et al., 2019). Hence, it is reasonable to expect that considering the tissue and cell type context of co-expression RNA-seq data will improve the quality of such predictions. However, the use of manually annotated tissue and cell type-specific gene expression patterns poses practical limitations:

I. When making predictions about a specific annotation, the correct cellular context of the particular annotation has to be considered. With potentially hundreds of relevant tissues and cell types to choose from, tissue and cell type-specific predictions require significant domain knowledge.
II. While some gene expression resources are well organized into manually annotated tissues and cell types, for example, GTEx (Consortium, 2015) or TCGA (Tomczak, Czerwińska & Wiznerowicz, 2015), these resources only cover a fraction of all human tissues and cell types. On the other hand, resources that contain more diverse datasets such as ARCHS4 (Lachmann et al., 2018) lack accurate manual tissue and cell type classification of samples.
III. Gene annotations prediction algorithms that utilize gene-gene co-expression data are applied to predict thousands of annotations. Thus, manual input for each case is not feasible.

The Simpson’s paradox (Blyth, 1972) is a statistical phenomenon where the relationship between two variables is different when observed in different subgroups of a population, compared to when the entire population is considered. In the context of gene expression, correlations between pairs of genes will only be detectable in data from subgroups of cell types and tissues, not captured by measuring correlations over all samples. The Simpson’s Paradox is usually something to be avoided, but here, we take advantage of it to enrich the feature space for gene annotation predictions.

**Figure 1:**
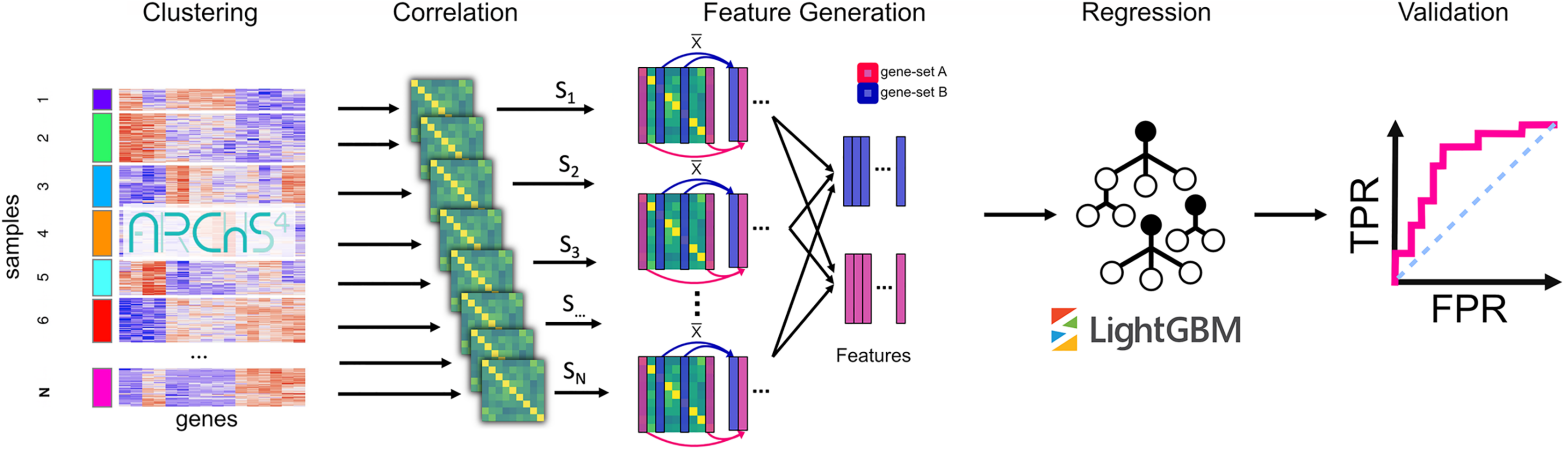
The PrismEXP workflow for gene annotation prediction. First, gene expression samples are clustered using k-means. For each cluster, a correlation matrix is computed. The prediction features for a gene $g$ and a gene set *GS* are computed as the average correlation of the gene *G* in the set *GS* for each correlation matrix individually. The features are then rearranged into a feature matrix for all genes, and a single gene set. These feature matrices are passed to a regression model using the LightGBM regressor. Finally, predicted gene annotations are validated using known annotations.

To apply an unsupervised machine learning approach called PRediction of gene Insights from Stratified Mammalian gene co-EXPression (PrismEXP) we generated a high dimensional feature space from large unlabeled RNA-seq mRNA gene expression data. The generated feature space automatically encodes tissue and cell type-specific information and produces tissue and cell type-specific correlation patterns, thus facilitating improved gene annotation predictions (Fig. 1). As demonstrated by computational experiments, the PrismEXP approach significantly improves gene annotation predictions compared to using the global gene-gene co-expression correlation matrix.

## Methods

### Data resources

To compute pairwise gene-gene mRNA co-expression correlations, we used the mouse ARCHS4 RNA-seq gene expression dataset (Version mouse matrix v2.1.2.h5, 10-19-2022). We chose mouse over human because benchmarking (Lachmann et al., 2018) demonstrated that predictions made with the mouse co-expression data slightly outperformed predictions derived from human co-expression data. The ARCHS4 mouse dataset is composed of 589,936 uniformly aligned RNA-seq gene expression samples publicly available from GEO (Clough & Barrett, 2016). For benchmarking, we utilized all 163 available gene-set libraries from Enrichr (Kuleshov et al., 2016). For more detailed analysis, we used six gene-set libraries from Enrichr (Table 1). In total, there are 14,169 annotated gene sets in these six libraries.

**Table 1:** Gene-set libraries from Enrichr with the number of annotated gene sets and unique genes. Literature-based gene-set libraries are manually curated, and non-literature-based libraries are derived directly from experimental data.

GMT		Description	Literature	Sets	Genes
ChEA 2016	Lachmann et al. (2010)	Transcription factor targets from ChIP-seq experiments	x	645	17,022
KEA 2015	Lachmann & Ma’ayan (2009)	Kinase-substrate interactions	x	428	2,904
MGI phenotype	Smith, Goldsmith & Eppig (2004)	Gene-phenotype associations from knock-out mice	x	5,261	10,390
hu.MAP	Drew et al. (2017)	Protein-protein interactions	c	995	2,117
GO: Biological process	Ashburner et al. (2000)	Manually curated gene functions	c	5,103	12,096
GWAS catalog 2019	Buniello et al. (2019)	Curated SNP-trait associations from GWAS studies	c	1,737	10,744

### Generating the gene-gene mRNA co-expression matrices

Genes with low gene expression are first filtered out, resulting in a set of genes, denoted as *G*. The threshold to include genes in *G* requires that a gene has at least 20 reads in 1% of all samples. This filtering step is intended to remove pseudo-genes and genes with low level of transcript abundance. PrismEXP automatically identifies a set of *N* clusters defined as *C* using a k-means clustering algorithm, where *N* is specified by the user. For each cluster, we apply quantile normalization on the log2 transformed gene counts to correct for library size variability. For each resulting normalized gene expression matrix, we calculate the pairwise gene-gene correlations for genes in *G* using the Pearson correlation coefficient method. Additionally, we calculate the Pearson correlation coefficients between genes across a random selection of 10,000 samples from all aligned samples in ARCHS4, ${C_{global}}$. The set of correlation coefficient matrices is: $C = \{ {C_0},{C_1}, \ldots ,{C_N},{C_{global}}\}$.

### Computing gene × gene-set features

Gene × gene-set features are scores for the strength of association between a gene and a gene-set. For a gene ${g_x} \in \{ {g_1}, \ldots ,{g_L}\}$ and a gene-set ${p_y} \in \{ {p_1}, \ldots ,{p_M}\}$ the PrismEXP algorithm generates a set of features ${\tilde S_k}$ used in the final prediction step. A gene-set library $S \in L \times M$ is defined as a binary matrix with genes as rows and gene-sets as columns. Such binary matrix can be represented as follows,

${S_{({g_x},{p_y})}} = \left\{ {\matrix{ {1,} & {{\rm{if}}\;{g_x} \in {p_y}} \cr {0,} & {{\rm{otherwise}}} \cr } } \right.$

Given *N* gene-gene co-expression matrices ${C_k} \in \{ {C_1}, \ldots ,{C_N}\}$ and a gene-set library *S*, the corresponding gene-annotation association features ${\tilde S_{({C_k},{g_x},{p_y})}}$ are defined as,

${\tilde S_{({C_k},{g_x},{p_y})}} = {{\sum\nolimits_{i = 1,i \ne x}^N {{S_{({g_x},{g_i})}} \times {C_{k({g_i},{p_y})}}} } \over {\sum\nolimits_{i = 1,i \ne x}^N {{C_{k({g_i},{p_y})}}} }}$

Alternatively, a gene-set library can also be defined as a non-binary adjacency matrix. Given *N* gene-gene co-expression matrices, each pair of gene and gene-set-annotation-term has the features,

${F_{({g_x},{p_y})}} = \{ {\tilde S_{({C_1},{g_x},{p_y})}}, \ldots ,{\tilde S_{({C_N},{g_x},{p_y})}}\} .$

### Training the regression model

PrismEXP takes the features ${F_{{g_x},{p_y}}}$ to classify whether ${g_x}$ should be annotated with ${p_y}$. Support Vector Machine (SVM), artificial neural networks (ANN), and other machine learning algorithms are suitable to apply for constructing predictive models in this context. PrismEXP utilizes the lightGBM regression model (Ke et al., 2017) to accomplish this task in our current implementation. The choice of lightGBM was made after testing several other machine learning models including SVM, multi-layer perceptron regressor, logistic regression, XGBoost, Gaussian naive Bayes, k-nearest neighbor, and linear discriminant analysis, and Random Forest (Suykens & Vandewalle, 1999; Murtagh, 1991; Kleinbaum et al., 2002; Chen & Guestrin, 2016; Rish, 2001; Indyk & Motwani, 1998; Balakrishnama & Ganapathiraju, 1998; Liaw & Wiener, 2002). All these regressors performed comparably. However, lightGBM performed slightly better than all\tested regressors, and also had the best performance with respect to compute time. The lightGBM model is trained with the following data,

$T = \{ ({x_1} = {F_{({g_{{x_1}}},{p_{{y_1}}})}},{y_1} = {S_{({g_{{x_1}}},{p_{{y_1}}})}}), \ldots ,({x_n} = {F_{({g_{{x_n}}},{p_{{y_n}}})}},{y_n} = {S_{({g_{{x_n}}},{p_{{y_n}}})}})\}$

### Python package and web-resources

PrismEXP is available as a Python package, a web-based interface, and an Appyter. The Python package supports programmatic prediction of gene annotations from gene expression data utilizing the data from ARCHS4 (Lachmann et al., 2018) stored in HDF5 format (Koranne, 2011). Predictions can be made for any gene-set library. The PrismEXP Python package also includes gene-set libraries to use directly from the collection of libraries available from Enrichr (Kuleshov et al., 2016). The PrismEXP Python package offers parameter customization. For example, the user can adjust the number of gene expression clusters *C* to partition the global expression matrix. For the web-based interface, we precomputed PrismEXP predictions with 100 gene expression clusters for 38,721 genes and eight gene-set libraries from Enrichr. These libraries are: ChEA 2022, GO Biological Process 2021, GWAS Catalog 2019, Human Phenotype Ontology (HPO), KEA 2015, KEGG 2021 Human, MGI Mammalian Phenotype Level 4 2021, and huMAP. The results from such predictions can be accessed through the PrismEXP web server available at: https://maayanlab.cloud/prismexp. In addition, PrismEXP is also available as an Appyter (https://appyters.maayanlab.cloud/PrismEXP/). Appyters convert Jupyter Notebooks to web-based bioinformatics applications (Clarke et al., 2021). The PrismEXP Appyter enables users to execute the PrismEXP computational pipeline using their own data in a cloud environment without the need to do any coding. The PrismEXP Appyter has access to 51 precomputed correlation matrices stored in Amazon Web Services (AWS) S3. The Appyter implementation of PrismEXP directly extracts the required correlation matrices data from the precomputed matrices. A user interface enables the selection of a gene to query, as well as the selection of any gene-set library for making predictions. Alternatively, users can upload their own custom gene-set libraries in GMT format.

### Validation

To assess the quality of the predictions made by PrismEXP, we asked how well PrismEXP predicts known gene annotations. The PrismEXP predictions are provided as a matrix with genes as the rows, and annotations as the columns. The higher a value in the matrix, the more likely the corresponding gene is associated with the respective annotation. Thus, we applied two ways to assess the quality such predictions. The first benchmarking method is to rank the values of each row in descending order, and the second is to rank the values of each column in descending order. Ranking the values for each row is equivalent of ranking gene annotations for each gene from likeliest to least likely. Since there is prior knowledge about the associations of genes with known annotations, these incidences are marked as true positives. These true positives are expected to be highly ranked. Annotations that were not previously known to be associated with a gene are considered false, and are expected to have lower ranks. PrismEXP calculates an area under the receiver operating characteristic (AUROC) curve for each gene. These AUROCs are used to compare the quality of the predictions by different methods, model parameters, and underlying datasets.

Alternatively, we can rank the genes for each annotation by the most likely to the least likely. Similarly, we can calculate the AUROCs by labeling the known associations as true positives. Both sets of AUROC scores, ranking gene for likely annotations, and ranking annotations for likely genes, are critical to consider when evaluating and validating various sources of data and model parameter settings.

## Results

### Performance and hardware requirements

PrismEXP is optimized to run on standard desktop computers utilizing multiple cores for the clustering and correlation calculations. Since PrismEXP is using large matrices, it has significant memory requirements. For a realistic use-case, using the ARCHS4 human gene-gene co-expression dataset, the memory requirement is 22 GB. PrismEXP was benchmarked using an Intel i7-8750H with six cores and 64 GB of RAM using Python 3.8.5. PrismEXP was also tested on an AWS r5ad.2xlarge instance with eight cores and 64 GB of RAM running Ubuntu. Both tests produce comparable performance (Fig. S1). The memory consumption and compute time from the five different stages of PrismEXP execution, training it with 100 gene-gene co-expression matrices, shows that gene filtering and sample clustering is completed in ~50 min (Fig. S1). Calculating the correlation matrices takes the longest time, 75 min or <1 min per cluster. Reducing or increasing the number of clusters affects run-time linearly based on the number of clusters. The prediction step depends on the number of genes, the number of gene sets, and the average gene set size of the gene-set library. These estimates were determined based on training applied using the Gene Ontology (GO) Biological Processes (BP) 2021 library from the Enrichr gene-set library catalog. The GO-BP gene-set library contains 5,103 gene sets, 14,433 genes, and has an average of 36 genes per set. Training the lightGBM model with 40,000 positive samples and 200,000 negative samples took about ~20 min to complete. The steps shown above have to be performed only once to initialize the prerequisite data for making multiple predictions. Custom gene-set libraries can be used to train the lightGBM regression model. The only step that needs to be performed each time for each gene-set library is the prediction step.

**Figure 2:**
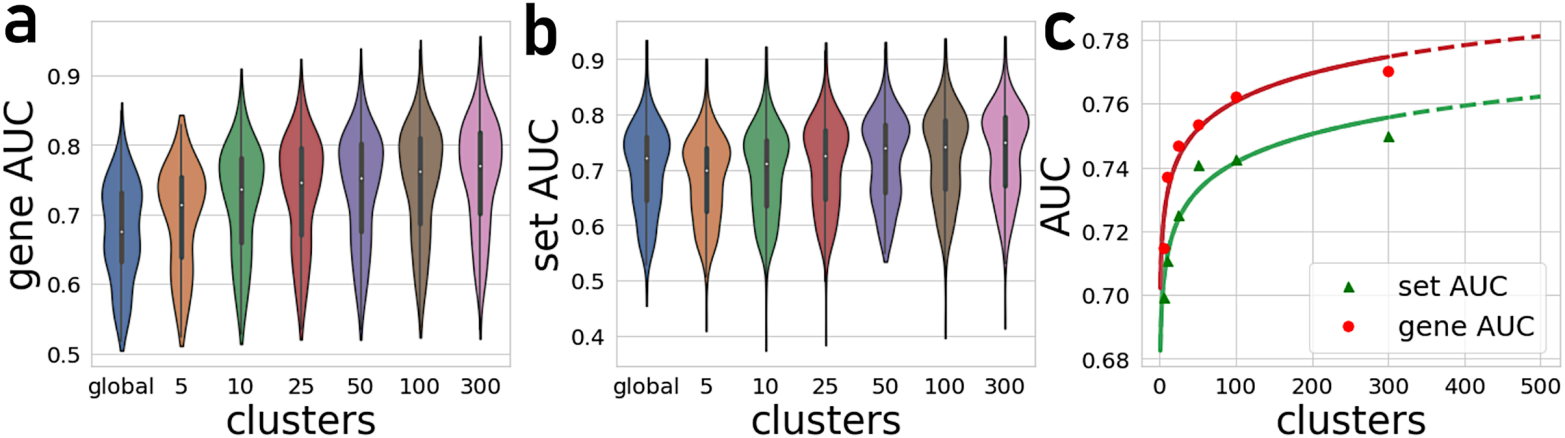
Average AUROCs of predicting known annotations for genes in gene sets from 163 gene-set libraries. (A) Average and distribution of AUROCs predicting known annotations for genes in gene sets as a function of the number of gene-gene co-expression matrices. (B) Average and distribution of AUROCs of gene predictions for the 163 gene-set libraries. (C) Curve fitting the AUROCs for gene and set predictions performance by different numbers of co-expression clusters.

The number of gene-gene co-expression matrices impacts the final prediction quality. We compared the prediction quality for partitioning the global matrix into 5, 10, 25, 50, 100, and 300 clusters (Fig. 2). These results are compared to predictions made with the global correlation matrix that is not partitioned. Overall, we observe a significant improvement in the average AUROCs when using the PrismEXP method over predictions made with the global co-expression correlation matrix. While the performance of the global correlation matrix outperforms PrismEXP for low numbers of clusters, PrismEXP significantly beats the prediction quality made by the global correlation matrix when more clusters are considered. We applied a paired sample Student t-test on the average AUROCs from 163 gene-set libraries to compare the overall performance of a single correlation matrix to applying PrismEXP with 301 clusters (Fig. 2). The results confirm significant improvement for using the partitioning approach, $p\lt{10^{ - 36}}$ and $p\lt{10^{ - 30}}$ for gene sets and genes, respectively. The improvement in average AUROCs achieved by increasing partitioning can be represented roughly by a logarithmic function of the form $f(x) = a \times {\log _2}(bx) + c$ (Fig. 2C). This fitting suggests that the number of clusters is logarithmically proportional to the average AUROCs. Increasing the number of clusters beyond 300 only marginally improves the predictions while increasing the computation time considerably. It is also expected that as some higher number of clusters the performance will begin to degrade. 

**Figure 3:**
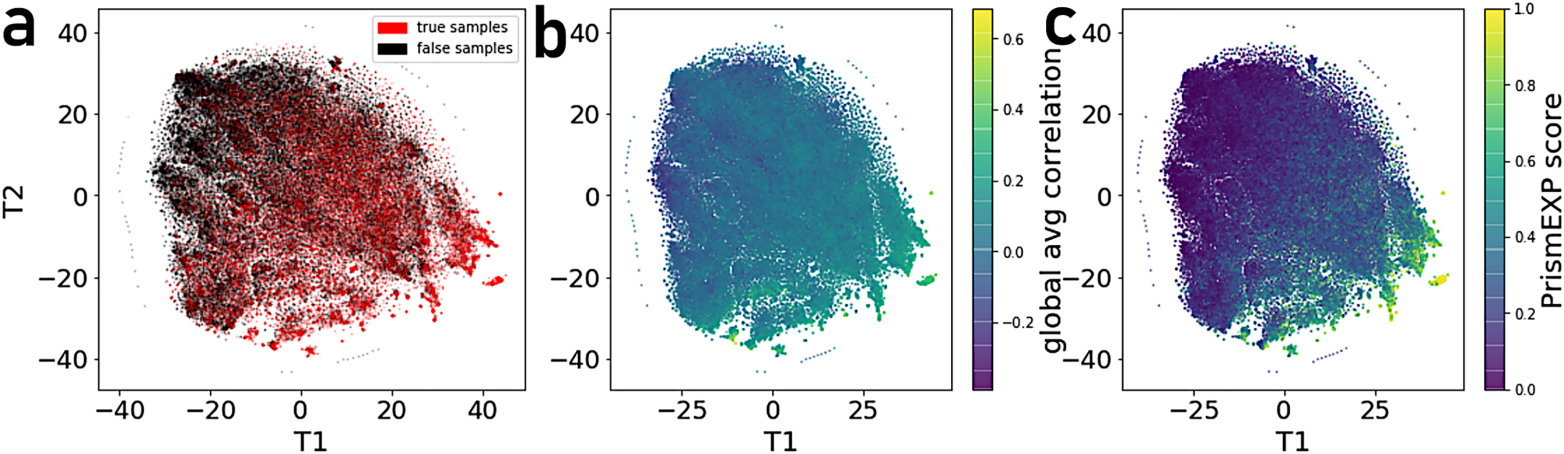
t-SNE visualization of 301 dimensional feature-space of 17,000 gene × gene-set pairs. (A) Distribution of gene × gene-set pairs with known associations (true positives). (B) Average correlation scores of gene × gene-set pairs relative to the high dimensional feature space. (C) PrismEXP scores for gene × gene-set pairs relative to the high dimensional feature space.

**Figure 4:**
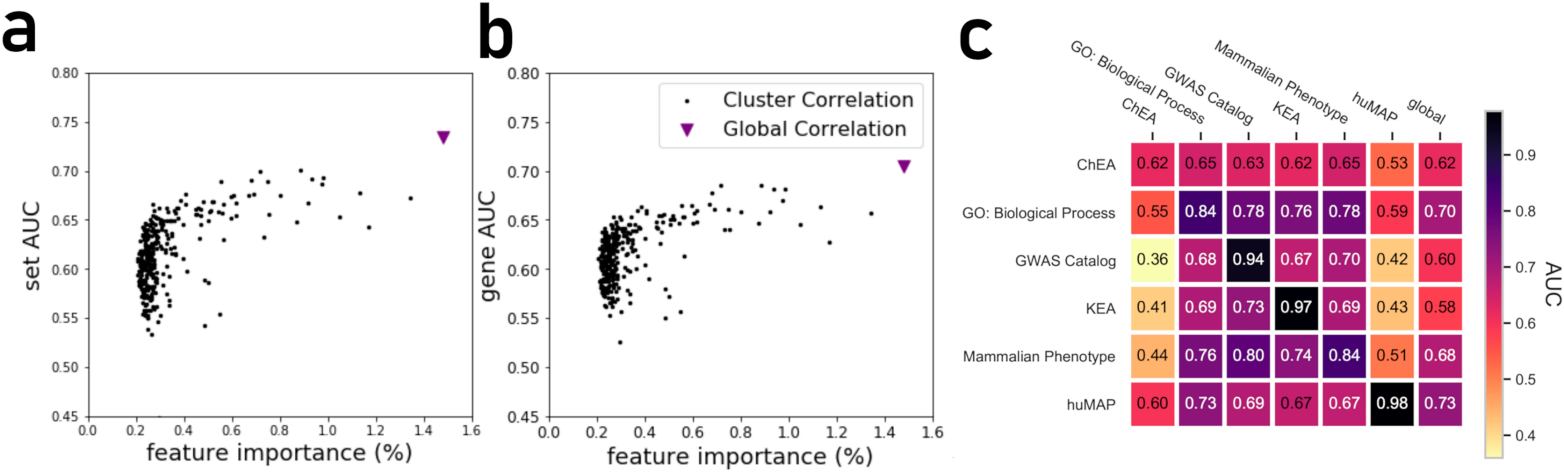
(A) Feature importance of individual correlation matrices relative to their prediction performance of ranking genes based on predicted known associations with biological functions. (B) Feature importance of individual correlation matrices relative to their prediction performance in ranking gene sets based on their associations with genes. (C) The PrismEXP lightGBM model prediction performance evaluated as the average gene AUROC when trained on six gene-set libraries and tested on the other libraries. The plot also includes prediction performance when using the global single correlation matrix.

By projecting the high dimensional feature-space of PrismEXP using t-SNE (Van der Maaten & Hinton, 2008) we observe distinct clusters (Fig. 3). In the first plot, each point represents a gene × gene-set pair associations, where known associations are marked in red (Fig. 3A). We observe that gene × gene-set pairs with prior known associations are organized into a gradient of average correlation from low on the left to high on the right (Fig. 3B). A similar gradient from left to right is observed for the PrismEXP predictions, following the true positives sample distribution (Fig. 3C). The features derived from the 301 co-expression correlation matrices, in combination with the Gene Ontology gene-set library as the training set, show that individual gene co-expression matrices contribute to the performance of the PrismEXP model (Figs. 4A and 4B). The feature importance of each co-expression correlation matrix is related to the prediction performance when using the average correlation of the correlation matrix alone. Co-expression correlation matrices that contribute a high level of feature importance, also perform better individually than matrices with low feature importance contribution. The global co-expression correlation matrix computed from 10,000 randomly selected samples extracted from the ARCHS4 resource has the highest single matrix performance and the highest feature importance contribution.

It is expected that a model trained with a specific gene-set library will produce high prediction performance when predicting gene annotations from that same library. However, we next asked how well a model trained with one gene library can predict terms from another library (Fig. 4C). A heatmap that compares average AUROCs across libraries illustrates that six PrismEXP models trained on one gene-set library listed in (Table 1) can be used to predict terms in other libraries. The prediction performance of a model trained on the same gene-set library is always the best, as expected. For example, a model trained on the hu.MAP gene-set library achieves an average gene AUROC of 0.98 when applied to make predictions for the same gene-set library. However, this model does not achieve good predictions for any of the other tested gene-set library. The results suggest that the hu.MAP gene-set library contains different co-expression patterns than the other libraries, and thus do not generalize gene annotation predictions across a diverse set of libraries. However, the model trained with GO Biological Processes (BP) achieves good prediction performance when used to predict other libraries’ functions. The GO-BP model also outperforms the single matrix predictions using all tested gene-set libraries, suggesting that the GO-BP model is generalizable across multiple gene-set libraries (Fig. 4C).

### Predicting gene associations to MGI mouse phenotypes

**Figure 5:**
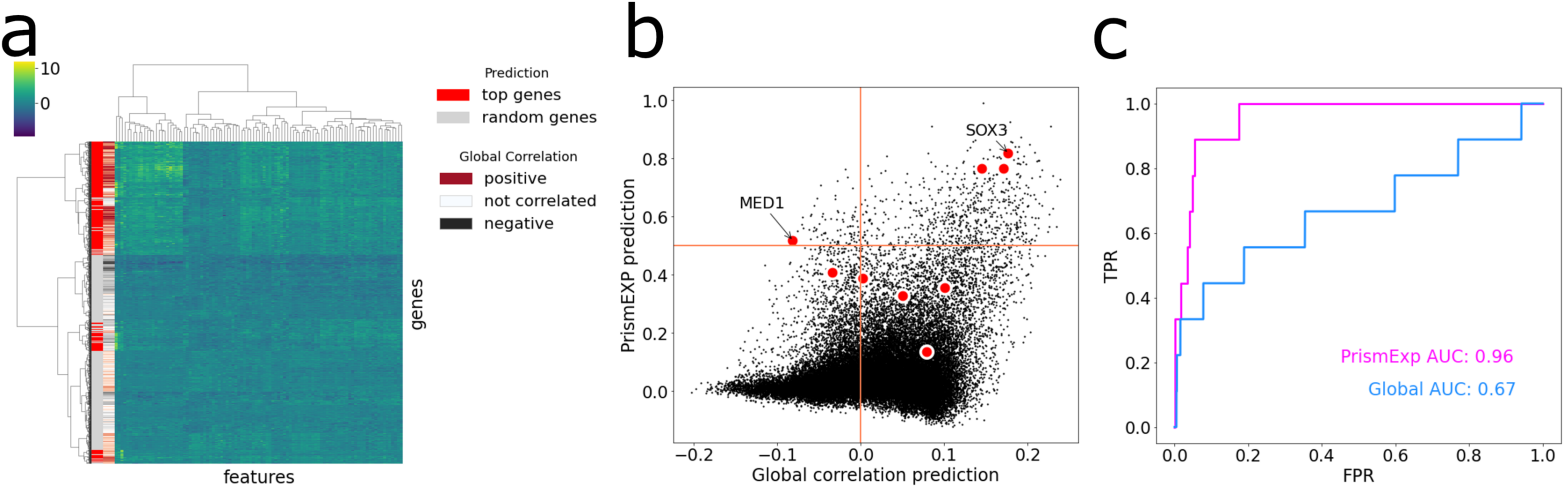
Feature space of the top predicted genes for MGI Mouse Phenotype MP:0005134 Decreased Thyroid-Stimulating Hormone Level. (A) Cluster heat map of the top predicted genes (colored in red) and 1,000 randomly selected genes (colored in grey) in the feature space. Values are plotted by z-score transformed feature values and clustered based on Euclidean distance using Ward’s linkage method. The subplot also highlights the average correlations between genes in the gene set MP:0005134. (B) Scatter plot of the average correlation between genes in the gene set MP:0005134. On the x-axis are the PrismEXP prediction scores and on the y-axis are the prediction with the global matrix. Genes annotated with MP:0005134 are highlighted in red. (C) Comparing the performance of the two methods in predicting genes associated with the MGI Mouse Phenotype MP:0005134 Decreased Thyroid-Stimulating Hormone Level.

The MGI Phenotype Database is a comprehensive resource for characterizing single gene knockout phenotypes in mice (Law & Shaw, 2018). PrismEXP can predict mouse phenotypes for genes with a high level of accuracy, specifically, with an average AUC of 0.82. To demonstrate this, we visualize the feature space of the top predicted genes for the MGI Mouse Phenotype MP:0005134 Decreased Thyroid-Stimulating Hormone Level (Fig. 5). When we hierarchically cluster the genes based on their feature similarities, we observe that the top predicted genes are not always positively correlated with the gene set, and different clusters of the feature space can be predicted as relevant in the context (Fig. 5A). The gene ranks by PrismEXP diverge from those computed by the global correlation matrix (Fig. 5B). Here, we highlight the annotated genes of MP:0005134. Two genes in particular, MED1 and SOX3, exhibit different relationships with the other genes in the gene set. SOX3 has a high average correlation to the annotated genes in MP:0005134 using the global correlation matrix, and a high PrismEXP score. On the other hand, MED1 has a slightly negative correlation when examined with the global correlation matrix, seemingly unrelated to the annotated genes in the set. However, by taking the whole feature space into account, PrismEXP is able to rank MED1 highly in this context. Overall, the prediction accuracy for this annotated gene set is significantly improved by the PrismEXP method from 0.67 with the global matrix to 0.97 with PrismEXP (Fig. 5C).

### Predicting under-studied druggable targets with PrismEXP

The Illuminating the Druggable Genome (IDG) project is focused on furthering our knowledge about understudied potential protein targets from the ion channel, kinase, and GPCR gene families (Oprea et al., 2018). Here we applied PrismEXP to predict annotations for one representative understudied member from each target family. The most significant associations for the protein kinase alpha-kinase 3 (ALPK3) are illustrated in a network diagram that connects ALPK3 with the top predicted GO terms and other relevant genes (Fig. S2A). As of December 2022, there were only 35 publications that mention ALPK3 on PubMed. One of these reported that biallelic loss of function of this understudied kinase is implicated in early-onset cardiomyopathy (Al Senaidi et al., 2019). The top annotations predicted for ALPK3 by PrismEXP are skeletal muscle contraction ( $p \;{<}\;{10^{ - 15}}$), myosin filament assembly ( $p \;{<}\;{10^{ - 14}}$), cardiac muscle hypertrophy ( $p \;{<}\;{10^{ - 13}}$), cardiac muscle fiber development ( $p \;{<}\;{10^{ - 13}}$), and striated muscle contraction ( $p \;{<}\;{10^{ - 12}}$), supporting the potential involvement of this kinase in cardio-pathologies. In addition, ALPK3 is highly related to well-studied genes that form the sarcomere, namely TTN, MYOM1, MYOM2, and MYOM3. Hence, the understudied kinase ALPK3 likely has an important role in cardiac function that is currently under-appreciated, potentially by phosphorylating some of these co-expressed targets. For the GPCR GPR183, PrismEXP predicts involvement in immune system-related processes. The top five terms are B-cell activation ( $p \;{<}\;{10^{ - 15}}$), lymphocyte chemotaxis ( $p \;{<}\;{10^{ - 9}}$), positive regulation of B-cell proliferation ( $p \;{<}\;{10^{ - 9}}$), T-Cell differentiation ( $p \;{<}\;{10^{ - 9}}$), and T-Cell activation ( $p \;{<}\;{10^{ - 8}}$). The genes that are highly associated with GPR183 are the immune-response cytokines IL2, IL4, and CCL19 (Fig. S2B), and the predicted associated kinases for GPR183 are the non-receptor tyrosine kinases LCK, TXK, BLK, and ITK. Hence, GPR183 activity is likely directly or indirectly related to mechanisms of immunity. Hence, it is possible that GPR183 acts as an upstream transmembrane sensor that regulates the activity of these kinases in immune cells. Finally, for the ion channel CLIC5, PrismEXP predicts a role in the inner ear including annotations such as receptor stereocilium organization ( $p \;{<}\;{10^{ - 20}}$), sensory perception of mechanical stimulus ( $p \;{<}\;{10^{ - 7}}$), regulation of systemic arterial blood pressure ( $p \;{<}\;{10^{ - 6}}$), vasoconstriction ( $p \;{<}\;{10^{ - 5}}$), and positive regulation of vasculogenesis ( $p \;{<}\;{10^{ - 5}}$) (Fig. S2C). Loss of CLIC5 has been associated with deafness (Seco et al., 2015). Hence, PrismEXP can be used as a predictive resource for illuminating knowledge about under-studied genes and proteins in the IDG focused families and beyond.

## Discussion

The predictions produced by PrismEXP can be used to stimulate hypothesis generation and guide the design of functional wet-bench experiments. Specifically, PrismEXP generates ranks for all human genes based on annotated gene sets, including over 400,000 annotated gene sets from Enrichr (Kuleshov et al., 2016), or those that are provided by a user. If a gene of interest is already annotated with a disease or a pathway, PrismEXP can be used to highlight other genes that might be missing from these annotations.

As with all machine learning applications in this domain, there is a risk of over-fitting the PrismEXP model to patterns embedded within one specific gene-set library used for training. Hence, we evaluated the performance of models trained to predict annotations for one library, to make annotation predictions for another unrelated library. We confirmed that the models are, in general, generalizable. In addition, we observed a significant improvement in the predictions for hu.MAP and KEA due to the partitioning of the samples for implementing the PrismEXP approach. One possibility to explain this observation is that protein-protein interactions and kinase-substrate phosphorylation reactions are highly context-specific.

It should be highlighted that all the predictions made by PrismEXP were made with features derived from RNA-seq mRNA gene-gene co-expression data. However, other omics data such as those from proteomics and epigenomics could be incorporated into the same framework. By adding other sources of gene-gene similarity, additional features can be added to the model without much modification. We have explored the use of other gene-gene similarity matrices for annotation predictions for other applications. For example, Geneshot (Lachmann et al., 2019) contains predicted gene annotations with features from gene-gene co-mentions in the literature, and gene-gene co-occurrences from gene-set queries submitted to Enrichr (Kuleshov et al., 2016). However, we never attempted to partition these gene-gene similarity matrices created from these other sources.

Finally, to make the PrismEXP algorithm accessible to the community, we developed a Python package that enables computational biologists to access the PrismEXP method programmatically. In addition, we also provide access to the predictions that are made by PrismEXP *via* a dedicated website and an Appyter. The Appyter and webserver application provide access to gene annotation predictions for users without programming skills. In the future, we plan to add the PrismEXP predictions to the ARCHS4 (Lachmann et al., 2018) gene pages.

## Conclusions

Here we show that by automatically partitioning the ARCHS4 gene-gene co-expression correlation matrix we can significantly improve gene annotation predictions from co-expression data. Such automated partitioning is likely positively correlated with tissue and cell type-specific partitioning, but without the need for manual metadata labeling. The method treats each gene expression cluster as a source for features to construct unsupervised machine learning models for gene annotation prediction. This approach is different from prior work that requires correct labeling of the gene expression profiles, for example, by text mining the metadata (Greene et al., 2015). Another advantage of PrismEXP is that the RNA-seq data is available for all genes. Thus, it is suitable for predicting annotations for understudied genes, including non-coding genes and splice variants.

## Supplemental Information

10.7717/peerj.14927/supp-1 Supplemental Information 1 Supplemental Figures.

## Additional Information and Declarations

### Data Availability

The following information was supplied regarding data availability: The PrismEXP web-based application, with pre-computed PrismEXP predictions, is available at: https://maayanlab.cloud/prismexp. PrismEXP is also available as an Appyter: https://appyters.maayanlab.cloud/PrismEXP/, and a Python package: https://github.com/maayanlab/prismexp. The code is available at Zenodo: Lachmann, Alexander, Rizzo, Kaeli, Bartal, Alon, Jeon, Minji, Clarke, Daniel J. B., & Ma’ayan, Avi. (2023). PrismEXP: gene annotation prediction from stratified gene-gene co-expression matrices (1.86). Zenodo. https://doi.org/10.5281/zenodo.7501923.
