# Supplementary material for: PrismEXP: gene annotation prediction from stratified gene-gene co-expression matrices
Source: PeerJ. 2023 Feb 27;11:e14927. doi: 10.7717/peerj.14927 (PMC9979837; doi:10.7717/peerj.14927)
Supplement: Supplemental Information 1 [file peerj-11-14927-s001.pdf]

SUPPLEMENTARY FIGURES

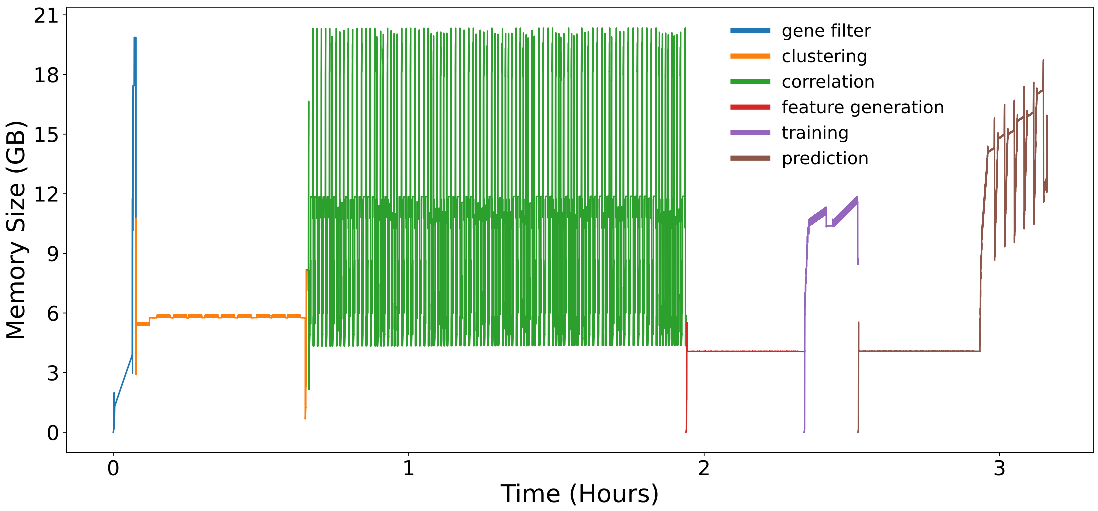

Figure S1. Memory utilization and compute time of PrismExp for 100 gene expression clusters and 27411 genes passing the gene filter step. Training was performed on training data derived from Gene Ontology Biological Process 2021 library using 240,000 samples. The compute steps of gene filter, clustering, correlation, feature generation, and training are pre-computation steps which only need to be run once. In the prediction step Biological Process annotations were predicted for all 27,411 genes

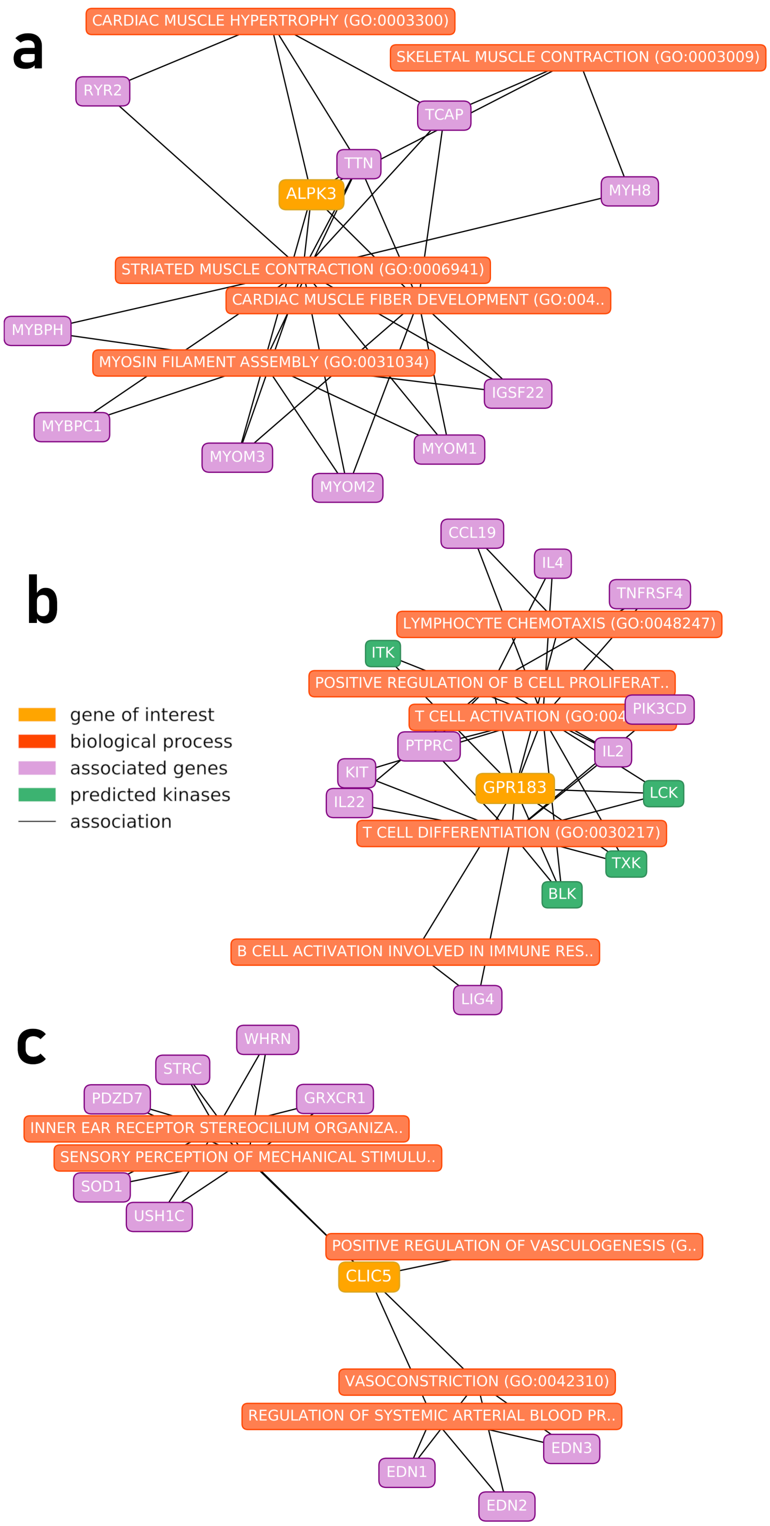

Figure S2. Top 5 predicted Gene Ontology Biological Processes (orange) and most associated other genes (purple) for understudied targets (yellow) **a)** The protein kinase ALPK3, **b)** The GPCR GPR183, and **c)** the ion channel CLIC5. Green nodes represent predicted kinases for GPR183.
